# Unfolded protein response in balancing plant growth and stress tolerance

**DOI:** 10.3389/fpls.2022.1019414

**Published:** 2022-10-07

**Authors:** Yao Liu, Yonglun Lv, An Wei, Mujin Guo, Yanjie Li, Jiaojiao Wang, Xinhua Wang, Yan Bao

**Affiliations:** ^1^ School of Agriculture and Biology, Shanghai Jiao Tong University, Shanghai, China; ^2^ Shanghai Collaborative Innovation Center of Agri-Seeds, Joint Center for Single Cell Biology, Shanghai Jiao Tong University, Shanghai, China

**Keywords:** UPR – unfolded protein response, ER stress, IRE1, bZIP, pollen, root

## Abstract

The ER (endoplasmic reticulum) is the largest membrane-bound multifunctional organelle in eukaryotic cells, serving particularly important in protein synthesis, modification, folding and transport. UPR (unfolded protein response) is one of the systematized strategies that eukaryotic cells employ for responding to ER stress, a condition represents the processing capability of ER is overwhelmed and stressed. UPR is usually triggered when the protein folding capacity of ER is overloaded, and indeed, mounting studies were focused on the stress responding side of UPR. In plants, beyond stress response, accumulating evidence suggests that UPR is essential for growth and development, and more importantly, the necessity of UPR in this regard requires its endogenous basal activation even without stress. Then plants must have to fine tune the activation level of UPR pathway for balancing growth and stress response. In this review, we summarized the recent progresses in plant UPR, centering on its role in controlling plant reproduction and root growth, and lay out some outstanding questions to be addressed in the future.

## What is UPR?

ER stress response refers to an action of protective mechanism, which can be triggered by various environmental stresses or protein folding problems in the ER. In order to cope with ER stress and maintain ER homeostasis, an efficient mechanism termed ERQC (ER quality control) is configured for ER stress response (Chen et al., 2020; Sun et al., 2021). Eukaryotic cells repair or clear damaged proteins through a series of ERQC systems to maintain homeostasis in the ER. UPR (unfolded protein response) is one of the key ERQC strategies that can effectively respond to ER stress. UPR is highly conserved among eukaryotes, and during ER stress, UPR modulates downstream targets to increase the folding capacity of ER and protect the organism from stress. In metazoans, the three canonical UPR sensors IRE1 (Inositol-requiring enzyme 1), ATF6 (Activating transcription factor 6) and PERK (Protein kinase R (PKR)-like ER kinase) are ER membrane localized, and have the ability to sense unfolded or misfolded proteins in ER and transduce downstream UPR signals (Doultsinos et al., 2017). Type I membrane proteins include IRE1 and PERK, which contain an N-terminal ER lumenal sensing domain, a transmembrane domain, and a C-terminal cytosolic domain for signal output. While ATF6 belongs to a type II membrane protein, which is structurally similar to type I membrane protein except that its N-terminus is in the cytosol and the C-terminus in the ER lumen. Alternatively, in other events, ER stress signals can also be transduced *via* noncanonical UPR pathways, independent of the three classic UPR sensors (Yu et al., 2022).

## Two branches of canonical UPR in plants

**Figure 1:**
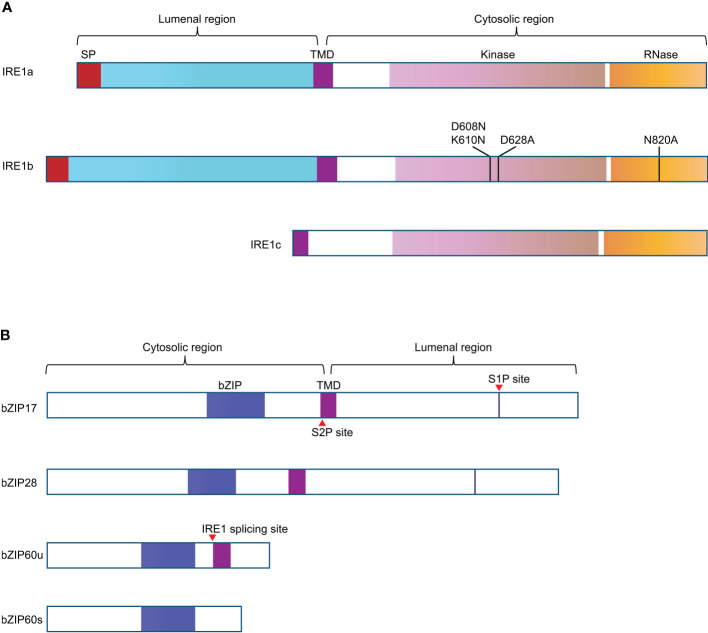
Schematic representation of the structural features of the UPR signal transducers in Arabidopsis. **(A)** A transmembrane domain (TMD) divides IRE1a and IRE1b into two segments. The N-terminal region of IRE1 has a signal peptide (SP, in red) and a sensing domain, facing the ER lumen. The C-terminal region of IRE1 contains the protein kinase domain (in light purple) and RNase domain (in orange), facing the cytosol. The mutation D608N K610N in IRE1b was supposed to be able to block its nucleotide binding activity, D628A mutation in IRE1b can interfere with the protein kinase activity of IRE1b, and N820A mutation knocks out the RNase activity of IRE1b. IRE1c is plant unique as it only has the corresponding TMD and cytosolic region. **(B)** bZIP transcriptional factors of UPR pathway have N-term cytosolic bZIP domain and C-term luminal region. The blue boxes represent bZIP domains, the purple boxes indicate the TMD, the red triangle arrows represent the corresponding cleavage or splicing site. bZIP60s (active form) and bZIP60u are spliced and unspliced forms of bZIP60.

**Figure 2:**
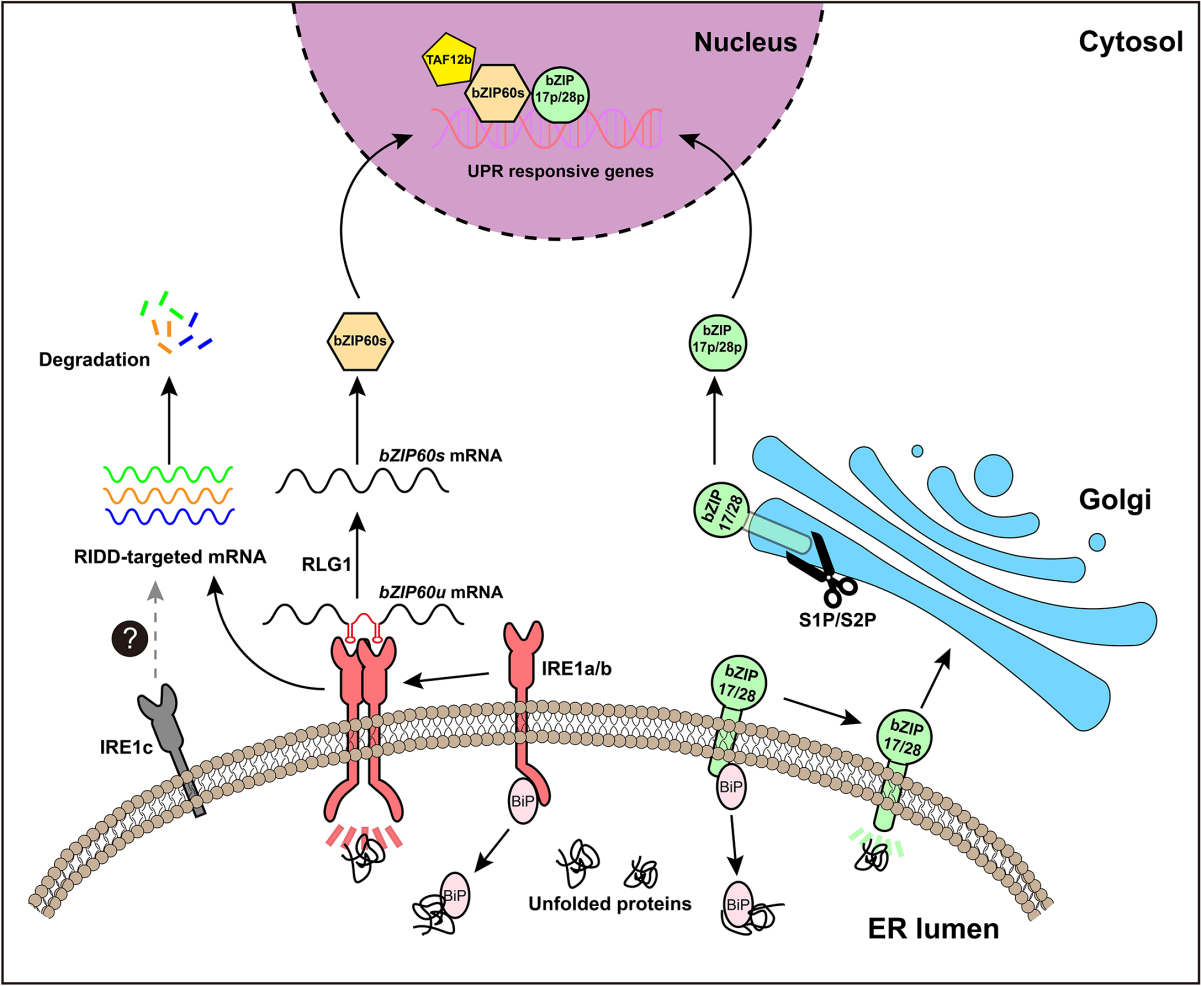
An overview of UPR signaling pathway in Arabidopsis. The plant UPR signaling pathway has two branches. The first branch is mediated by IRE1a and IRE1b. Under ER stress, the chaperone protein BiP, binds to unfolded proteins, thereby releasing IRE1 to form an active dimeric or polymeric form. Activated IRE1 splices the mRNA of *bZIP60u* to produce *bZIP60s*, releasing a hairpin RNA (in red). The spliced fragments of bZIP60 mRNA will be reunited by t-RNA ligase RLG1. *bZIP60s* mRNA encodes the active transcription factor bZIP60s, which can enter the nucleus to induce the expression of related UPR target genes. In addition, activated IRE1 is involved in another process, known as RIDD (regulated IRE1-dependent decay), which degrades other mRNAs and ultimately reduces the synthesis of new proteins. The second branch is mediated by bZIP17 and bZIP28, two ER membrane-localized transcription factors. Under ER stress conditions, bZIP17 and/or bZIP28 are relocated from the ER to the Golgi, where they are hydrolyzed by S1P and S2P protease, and subsequently their hydrolyzed TF domains (bZIP17p and/or bZIP28p) are released for further importing to the nucleus to regulate the expression of ER stress responsive genes. IRE1c is a recently discovered plant-specific IRE1 subtype, but the exact function of IRE1c and its role in plant UPR pathway need to be further examined.

No clear PERK homologs were found in plants and, to date, only two branches of plant UPR pathway have been discovered. In Arabidopsis, the first branch of UPR is mediated by AtIRE1a and AtIRE1b (**Figure 1A**), two homologues of metazoan IRE1, which are close in protein structure and function (Koizumi et al., 2001; Noh, 2002). During ER stress, AtIRE1a and AtIRE1b work redundantly to cleave the mRNA of basic leucine zipper family member *bZIP60* (*bZIP60u*) with unconventional splicing. Post to the cleavage of 23 bp (base pair) nucleotides from *bZIP60u*, a t-RNA ligase called RLG1 will re-join the two segments and produce *bZIP60s* (spliced form of *bZIP60* mRNA) (Nagashima et al., 2016). Spliced *bZIP60s* encodes the active form of bZIP60 transcription factor, which can move into the nucleus to induce the expression of target genes (Iwata et al., 2008; Deng et al., 2011; Nagashima et al., 2011). In addition, under ER stress, the activated IRE1 also targets other mRNAs and degrades them, ultimately inhibiting the synthesis of new proteins, a process termed RIDD (regulated IRE1-dependent RNA decay) (**Figure 2**) (Mishiba et al., 2013).

The second branch of Arabidopsis UPR is carried out by membrane-associated transcription factors AtbZIP17 and AtbZIP28 (**Figure 1B**), which are functional homologs of metazoan ATF6 (Liu et al., 2007a; Liu et al., 2007b). ATF6 confers ER localization through its transmembrane domain (Haze et al., 1999). In plants, Arabidopsis AtbZIP17 and AtbZIP28 are activated in similar ways to that of ATF6. Under ER stress conditions, ER-localized AtbZIP17 and AtbZIP28 were induced to transport to the Golgi, where they are sequentially hydrolyzed by S1P (site 1 protease) and S2P (site 2 protease) (Kinch et al., 2006), freeing the AtbZIP17p and/or AtbZIP28p to function as transcription factors in the nucleus (**Figure 2**). However, it has been demonstrated that activation of Arabidopsis bZIP28 is mediated by S2P rather than S1P (Iwata et al., 2017), thus, the underlying basis for this sequential activation needs further investigation.

The function of canonical UPR under ER stress has been extensively studied. However, in recent years, the research of non-canonical UPR pathways in plant growth and stress responding has also been intensified. In addition to bZIP family transcriptional factors, plant unique NAC (No apical meristem (NAM), Arabidopsis transcription activation factor (ATAF), and Cup-shaped cotyledon (CUC)) family transcriptional factors are also involved in UPR response. To date, three NAC transcription factors AtNAC062 (plasma membrane tethered), AtNAC089 (ER-localized) and AtNAC103 (cytosolic) were reported to be implicated in ER stress response via the regulation of UPR responsive genes in Arabidopsis (Sun et al., 2013; Yang et al., 2014a; Yang et al., 2014b).

Beyond transcriptional level of regulation, a variety of strategies were employed for noncanonical UPR responding. For instance, study of plant-specific, PIP binding SVB (Small Trichomes with Variable Branches) family proteins suggests a connection between PIP signaling and UPR response (Marks et al., 2009; Oxley et al., 2013; Yu and Kanehara, 2020; Yu et al., 2021). In addition, plant G protein signaling participates in ER stress response via collaboration with IRE1 (Wang et al., 2007; Chen and Brandizzi, 2012; Afrin et al., 2022). Arabidopsis BLISTER (BLI) protein affects plant growth via negative regulation of IRE1 (Hong et al., 2019), while IRE1-mediated ER stress signal output in rice is negatively regulated by SQUAMOSA PROMOTER-BINDING PROTEIN-LIKE 6 (SPL6) (Wang et al., 2018). Recently, UPR-related BiP2 and ERDJ3a were detected in SGs (Stress Granules), suggesting the potential of SG formation in UPR regulation (Advani and Ivanov, 2020; Maruri-Lopez et al., 2021).

## UPR in plant stress response

As stated above, accumulation of misfolded proteins in the ER can activate UPR pathway, which in turn regulates the expression of UPR responsive genes and helps to maintain ER homeostasis. Both environmental stresses and genetic disorders can cause misfolded proteins to trigger UPR. Protein folding is a delicate and complex process that is influenced by a variety of environmental factors. For example, salt, heat, drought and some biotic stresses all provoke UPR in plants. The studies regarding UPR in plant stress response have been extensively discussed (Howell, 2017; Manghwar and Li, 2022), which will not be the focus of this review.

## UPR in regulating plant root growth

**Figure 3:**
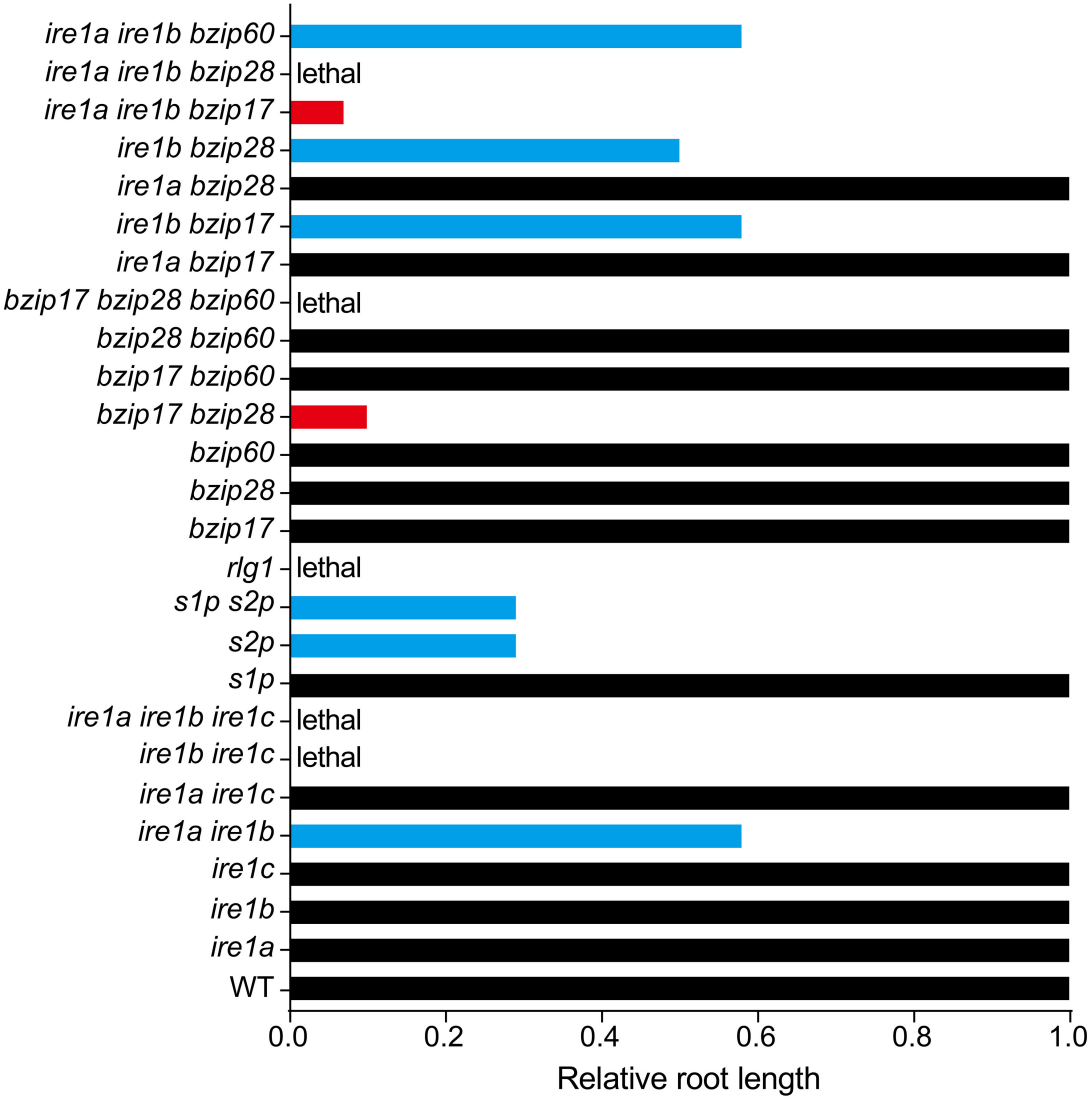
Relative root length of Arabidopsis canonical UPR pathway mutants. Root length of WT is set as 1.0, and the black columns indicate the root growth of relevant UPR mutants are not significantly affected. The blue columns represent about 50% reduction of root length in the relevant mutants. The red columns show stunted root length in two triple knock-out mutants *17ab* (*ire1a ire1b bzip17*, ~5% of WT) and *bzip17 bzip28* (*bz1728*, ~10% of WT). Data shown in this figure are based on published results regarding the root length of relevant UPR mutants compared with their corresponding WT.

Accumulating evidence suggests that plant UPR pathway components are closely involved in regulating plant root growth. Single Arabidopsis UPR mutants (*ire1a, ire1b, bzip17, bzip28, bzip60*) do not affect primary root growth under normal condition, but high order UPR mutants have additive effect on limiting primary root elongation (**Figure 3**). Compared with wild-type, the *ire1a ire1b* double mutant shows shorter primary root, and this effect is exemplified after DTT (Dithiothreitol, a redox reagent) or Tm (Tunicamycin, an inhibitor of N-linked glycosylation) treatment (Deng et al., 2013; Chen et al., 2014). IRE1-regulated root growth seems independent of its classic target *bZIP60*, as *bzip60* mutants act the same as wild-type in controlling primary root growth with or without ER stress. Moreover, introducing *bzip60* into *ire1a ire1b* background does not further reduce the root length of *ire1a ire1b*, again suggesting a limited role of bZIP60 in this process.

The other arm of UPR is the S1P/S2P-mediated proteolytic cleavage of bZIP17 and bZIP28. Single mutants of *s1p* have little effect on primary root elongation under non-stress condition, just like the single mutant *bzip17* or *bzip28*. However, *s1p* shows shorter root under salt stress when compared with that of the corresponding wild-type (Liu et al., 2007b). The single mutants of *s2p* have a profound effect on limiting primary root elongation under normal conditions, retaining its root length 30% of the wild-type, while the shoot of *s2p* is not significantly affected (Che et al., 2010). In addition, *s1p s2p* double mutant behaves a similar root growth rate to that of the *s2p* under normal condition (Kim et al., 2018), suggesting a major role of S2P in controlling root growth. The *bzip17 bzip28* double mutant exhibits stunted short root phenotype, even shorter than that of *s2p* or *s1p s2p*, along with dwarfed plant and delayed germination, indicating there is an unknown S1P/S2P-independent path for controlling bZIP17/28-mediated root growth. In the recent study, Kim et al. (2022) further found that NOBIRO6/TAF12b (TBP-ASSOCIATED FACTOR 12b (TAF12b)), a general transcription factor component, may function as a cofactor of bZIP60 for maintaining favorable level of UPR signal output. Regarding the rate of root recovery in *nobiro6 bzip17 bzip28*, bZIP60-independent pathway for controlling root growth is still yet to be identified.

Although each arm of plant UPR plays specific functions in certain pathways or conditions, both arms also have overlapping functions. For instance, bZIP28 works cooperatively with IRE1 and bZIP60 in ER stress responses (Liu and Howell, 2010; Howell, 2013). The *ire1b bzip28* double mutant exhibits a phenotype similar to that of the *ire1a ire1b* double mutant (**Figure 3**), inhibiting primary root elongation under both stress and non-stress conditions. Interestingly, the *bzip28 bzip60* double mutant does not affect root elongation under normal conditions. However, under ER stress, the *bzip28 bzip60* double mutant inhibits primary root elongation profoundly. As bZIP60 is a direct and classic target of IRE1, therefore, the author believes that the promoting effect of IRE1b on roots is unrelated to bZIP60 under non-stress conditions (Deng et al., 2013). bZIP17 was shown to function redundantly with bZIP28 in controlling plant root growth (Kim et al., 2018), but when combined with b*zip60*, the relevant *bzip17 bzip60* double mutant does not interfere with the elongation of primary root under non-stress condition. However, during ER stress, the primary root of *bzip17 bzip60* is significantly reduced, but not as profound as that of *bzip28 bzip60* (Deng et al., 2013; Kim et al., 2018; Bao et al., 2019). The primary root of *ire1b bzip17* is also significantly shorter than that of the wild-type, but this difference was neither observed in *ire1a bzip17* nor in *ire1a bzip28* (Bao et al., 2019). *ire1a ire1b bzip17* (*17ab*) triple mutant is viable and has very stunted primary root (5% of that in the wild-type, shorter than that of *bzip17 bzip28*), while *ire1a ire1b bzip28* is lethal. All above discoveries suggest a major role of IRE1b and bZIP28 in UPR-regulated root growth. Of note, *bzip17 bzip28 bzip60* triple mutant is not obtainable (Kim et al., 2018), implying the high redundance and essential role of those three transcriptional factors in regulating plant growth.

As mentioned above, elongation of primary roots under non-stress conditions depends on IRE1. IRE1 is a dual protein kinase and RNase, therefore, it is necessary to explore the influence of its different specific functions on root growth. Using site-directed mutagenesis, Deng et al. (2013) showed that disrupting the nucleotide binding activity (D608N K610N) or RNase activity (N820A) of IRE1b cannot restore the stress sensitive phenotype of *ire1a ire1b*, while the IRE1b kinase-dead variant (D628A) and nonmutated form can. However, none of the three mutated forms of IRE1b can restore the *17ab* dwarf phenotype or its root growth defects, suggesting the impacts of corresponding domains in IRE1 may vary depends on different genetic backgrounds applied. More importantly, the active nucleus-targeted forms of bZIP17, bZIP28 and bZIP60 can all restore growth defects of *17ab*, but the Golgi-detained bZIP17G372A cannot, suggesting UPR-associated bZIP transcriptional factors have shared functions and need to be activated for sustaining root growth, even without stress (Bao et al., 2019). Interestingly, in the previous research, Liu et al. (2007b) reported that constitutive over expression of the active form of bZIP17 can inhibit plant growth in the wild-type. Opposite function of bZIP17 in different genetic backgrounds looks intriguing but may somehow reflect the dual role of UPR in balancing plant growth and stress response.

IRE1c is a recently discovered plant-specific isoform of IRE1 family. The root length of *ire1c* single mutant is similar to that of the wild-type under ER stress or normal conditions. *ire1a ire1c* behaves the same as *ire1c*, however, *ire1b ire1c* and *ire1a ire1b ire1c* are lethal (Pu et al., 2019). The underlying mechanism of IRE1c in controlling plant root growth and UPR signaling is puzzling and yet to be verified.

## UPR in controlling plant reproduction

Pollen abortion is one of the most influential factors that can cause crop yield reduction. A variety of genetic and environmental factors can affect the normal development of pollen in flowering plants, including high temperature, drought, high light and beyond. Studies have shown that the temperature requirements during plant development are very strict, and pollen development is very sensitive to high temperature. Some transcription factors and RNA splicing factors are closely involved in the UPR and play a vital role in pollen resistance to heat stress (**Table 1**). IRE1, a major component of the UPR pathway, relies on its ribonuclease activity for splicing mRNA to protect male fertility from heat stress (Deng et al., 2016). Arabidopsis *ire1a ire1b* double mutant has normal and vigorous pollen development under normal growth conditions, while during heat stress, the double mutant has defective growth and exhibits temperature-sensitive male sterility traits. Intriguingly, overexpression of *SEC31A*, a COPII membrane vesicle trafficking protein and potential target of UPR pathway, rescued male sterility in the *ire1a ire1b* double mutant (Deng et al., 2016). Not coincidentally and pollen development under high temperature stress is also closely linked to bZIP28 on the other arm of plant UPR. Arabidopsis *bzip28 bzip60* double mutant plants exhibit a hypersensitive phenotype to high temperature stress, such as shorter cuticles and reduced fertility (Zhang et al., 2017). In the *ire1b bzip28* double mutant, pollen development is also slightly affected. Homozygous *ire1a ire1b bzip28* triple mutant is not viable, but pollens from the *ire1a ire1b bzip28+/-* mutant plant showed approximately 50% pollen abortion. Thus, it is concluded that IRE1b protein kinase and RNase activity are essential for male gametophyte establishment (Deng et al., 2013). Compared with the wild-type, Arabidopsis *bzip17* mutant is found to be sensitive to heat stress in affecting silique length and fertility. ChIP-Seq (chromatin immuno-precipitation coupled with high-throughput sequencing) found that 1645 genes are direct target genes of bZIP17 under heat stress. Among them, 113 genes are up-regulated by bZIP17 in flowers under heat stress. These results demonstrate that bZIP17 also plays an important role in maintaining plant fertility under heat stress (Gao et al., 2021).

**Table 1:** Reproduction property of Arabidopsis canonical UPR pathway mutants.

Genotype (Gene ID)	Normal condition	Heat stress	Reference
*ire1a* (AT2G17520)	normal	\	Mishiba et al., 2019
*ire1b* (AT5G24360)	normal	\	Mishiba et al., 2019
*ire1c* (AT3G11870)	normal	\	Mishiba et al., 2019
*ire1a ire1b*	normal	defective pollen, low fertility	Deng et al., 2016; Mishiba et al., 2019
*ire1a ire1c*	normal	\	Mishiba et al., 2019
*ire1b ire1c+/-*	aborted pollen; homozygous lethal	\	Pu et al., 2019
*ire1a ire1b ire1c+/-*	aborted pollen; short stamens; homozygous lethal	\	Mishiba et al., 2019; Pu et al., 2019
*s1p* (AT5G19660)	\	\	Liu et al., 2007b
*s2p* (AT4G20310)	\	\	Kinch et al., 2006
*s1p s2p*	\	\	Che et al., 2010; Kim et al., 2018
*rlg1+/-* (AT1G07910)	homozygous lethal	homozygous lethal	Nagashima et al., 2016
*bzip17* (AT2G40950)	normal	low fertility	Gao et al., 2022
*bzip28* (AT3G10800)	normal	\	Liu et al., 2007a; Gao et al., 2008
*bzip60* (AT1G42990)	normal	\	Iwata et al., 2008; Deng et al., 2011
*bzip17 bzip28*	\	\	Kim et al., 2018
*bzip17 bzip60*	normal	\	Deng et al., 2013; Bao et al., 2019
*bzip28 bzip60*	normal	low fertility	Deng et al., 2013; Zhang et al., 2017
*bzip60 bzip17 bzip28+/-*	heteromorphic flower; short stamens; homozygous lethal	\	Kim et al., 2018
*ire1a bzip17*	normal	\	Bao et al., 2019
*ire1b bzip17*	normal	\	Bao et al., 2019
*ire1a bzip28*	normal	\	Bao et al., 2019
*ire1b bzip28*	normal	\	Deng et al., 2013; Bao et al., 2019
*ire1a ire1b bzip17*	\	\	Bao et al., 2019
*ire1a ire1b bzip28+/-*	reduced pollen viability; homozygous lethal	\	Deng et al., 2013
*ire1a ire1b bzip60*	normal	\	Deng et al., 2013

+/-, heterozygote; /, not investigated.

Previous studies have shown that BiP (Binding Protein) proteins function coordinately in well-developed pollen and pollen tubes. Pollens from *bip1 bip2* double mutant plants are normal, but pollens from *bip1 bip2 bip3* triple mutant are lethal (Maruyama et al., 2014). Arabidopsis HOP3 (HSP70/HSP90 organizing protein 3) is a BiP interacting cytosolic cochaperone protein, whose expression is both transcriptional and translationally regulated by IRE1 during ER stress. When knocked out, *hop3* shows defects in pollen development and inability to resist ER stress (Fernandez-Bautista et al., 2017). In line with the above findings, TMS1 (Thermosensitive Male Sterile 1), another heat shock protein, acts downstream of *bZIP28* and *bZIP60* during heat stress and function as physical partners of BiP proteins in sustaining protein folding capacity for plant thermotolerance (Ma et al., 2015).

As mentioned above, the pollen development process is closely linked to the fertilization process, and the normal development of the endosperm in flowering plants cannot be achieved without the UPR, and key regulatory genes from both arms of UPR pathways are also involved in the regulation of rice grain chalkiness (Vitale and Boston, 2008; Zhang et al., 2017; Sandhu et al., 2021). *OsbZIP60* (a homolog of *AtbZIP28*), an important regulator of heat stress and drought stress response in rice, also regulates rice grain chalking. Loss of *OsbZIP60* gene results in hyper ER stress sensitivity during endosperm development. In the *osbzip60* mutant, UPR-associated genes *OsbZIP50* (a homolog of *AtbZIP60*) and *OsBiP2/3/4/5* are overactivated in endosperm cells and lead to a severe grain chalkiness phenotype. In addition, OsbZIP50 also amplifies ER stress signal by promoting its own transcription. And overexpression of *OsBiP2/3/4/5* leads to different degrees of chalkiness in grains, respectively. This further suggests that UPR activation in *osbzip60* mutant is inseparable from OsbZIP50, and that OsbZIP60 balances endosperm development and grain chalkiness by suppressing the overexpression of OsbZIP50 (Hayashi et al., 2012; Wakasa et al., 2012; Yang et al., 2022). Moreover, OsbZIP60 activates the expression of *Chalk5*, a master effector gene that regulates chalkiness traits in rice. Chalk5 disrupts protein biosynthesis in the rice endosomal system by acting as a vacuolar H^+^ transport pyrophosphatase (V-PPase), and overexpression of *Chalk5* enhances the endosperm and grain chalkiness phenotypes (Li et al., 2014).

Recent studies revealed a unique plant IRE1 subtype, AtIRE1c, which is only found in *Brassica* species. IRE1c and IRE1b have unexpected functional coordination in controlling gametogenesis in Arabidopsis (**Table 1**), however, there is no clear evidence of IRE1c in modulating canonical UPR (Mishiba et al., 2019; Pu et al., 2019). Functional diversification of IRE1c and its exact role in UPR pathway thus warrants further explorations.

## Discussions and future perspectives

After decades of efforts committed in studying Arabidopsis and some other plant species, great progresses were achieved in our understanding of plant UPR pathway. Attributing to the sessile feature of plants, UPR pathway components and the relevant mechanism of activation have been extensively investigated in environment-derived ER stress responding. In recent years, more and more evidence suggests that UPR is strongly involved in plant growth and development. Studies from (Bao et al., 2019) clearly suggested that, even without stress, endogenous basal activation of UPR pathway is required for maintaining normal plant growth, functional UPR thus plays dual roles in controlling growth and stress responding. How plants manipulate the same set of UPR components and fine-tune the level of UPR activation for balancing growth and stress response is ambiguous, but the underlying mechanism will be exciting to explore. Like UPR, autophagy also plays dual roles in regulating plant growth and stress response. ER stress-mediated autophagy activation seems IRE1 dependent and bZIP60 independent (Bao et al., 2018), reinforcing the critical role of IRE1-associated RIDD pathway in this process. Decoding the exact function of relevant RIDD targets is challenging but will be helpful in dissecting the principles of RIDD-mediated UPR regulation. In addition to UPR and autophagy, other ERQC system like ERAD (ER-associated degradation) was also showed to be involved in plant growth and stress responding (Chen et al., 2020). How plants leverage and coordinate each pathway for maintaining ER homeostasis is a long outstanding but critical question to be addressed. IRE1c family proteins were found to be unique for *Brassicaceae* species, which lack an ER lumenal sensing domain (Mishiba et al., 2019). Essential role of IRE1c will appear when acting in combination with IRE1b (Pu et al., 2019), but how does IRE1c exert its function with IRE1b is quite interesting and definitely justified for further investigations. Persistent ER stress and ER stress recovery are recent new trends in studying plant UPR pathway (Ruberti and Brandizzi, 2018; Srivastava et al., 2018; Ko and Brandizzi, 2022a; Ko and Brandizzi, 2022b), interpreting the relevant working language in UPR pathway will advance our understanding of plant environment adaptions and accelerate the breeding of stress-resistant crops.

## Author contributions

YB concepted the topic of this manuscript with YLiu. YL and YLL drafted the manuscript with YB. YB revised the manuscript. All authors contributed to the article and approved the submitted version.

## Funding

This research is supported by China Agriculture Research System of MOF and MARA.

## Conflict of interest

The authors declare that the research was conducted in the absence of any commercial or financial relationships that could be construed as a potential conflict of interest.

## Publisher’s note

All claims expressed in this article are solely those of the authors and do not necessarily represent those of their affiliated organizations, or those of the publisher, the editors and the reviewers. Any product that may be evaluated in this article, or claim that may be made by its manufacturer, is not guaranteed or endorsed by the publisher.
